# Development of a Network RTK Positioning and Gravity-Surveying Application with Gravity Correction Using a Smartphone

**DOI:** 10.3390/s130708879

**Published:** 2013-07-12

**Authors:** Jinsoo Kim, Youngcheol Lee, Sungyeoul Cha, Chuluong Choi, Seongkyu Lee

**Affiliations:** 1 Spatial Information Institute, Pukyong National University, 45 Yongso-ro, Nam-gu, Busan 608-737, Korea; E-Mail: pknu9680@gmail.com; 2 Geologic Hazard & Industrial Resources Research Institute, Pusan National University, 63 Busandaehak-ro, Geumjeong-gu, Busan 609-735, Korea; E-Mail: geoyoung@pusan.ac.kr; 3 Department of Civil Engineering, Yangsan University, 321 Myeonggok-ro, Yangsan 626-740, Korea; E-Mail: ysusycha@gmail.com; 4 Department of Spatial Information Engineering, Pukyong National University, 45 Yongso-ro, Nam-gu, Busan 608-737, Korea; E-Mail: cuchoi@pknu.ac.kr

**Keywords:** gravity survey, smartphone, gravity correction, network RTK positioning

## Abstract

This paper proposes a smartphone-based network real-time kinematic (RTK) positioning and gravity-surveying application (app) that allows semi-real-time measurements using the built-in Bluetooth features of the smartphone and a third-generation or long-term evolution wireless device. The app was implemented on a single smartphone by integrating a global navigation satellite system (GNSS) controller, a laptop, and a field-note writing tool. The observation devices (*i.e.*, a GNSS receiver and relative gravimeter) functioned independently of this system. The app included a gravity module, which converted the measured relative gravity reading into an absolute gravity value according to tides; meter height; instrument drift correction; and network adjustments. The semi-real-time features of this app allowed data to be shared easily with other researchers. Moreover, the proposed smartphone-based gravity-survey app was easily adaptable to various locations and rough terrain due to its compact size.

## 1. Introduction

Gravity measurements for gravity anomalies, mostly conducted by relative measurement using springs, have a precision of 0.001 mGal [1,2] and require several minutes to dozens of minutes for measurements to be complete. The measured gravity value is stored in the internal memory of the device, which can then be by a computer. Current relative gravity meters are convenient compared with conventional analogue gravity meters, having a relative ease of use for calculating gravity disturbance rapidly using ellipsoidal height data provided by the global navigation satellite system (GNSS).

Although measurement times have been reduced considerably, the field notes used to measure gravity utilize analogue methods. These field notes record the station name, location, measured gravity, measurement time, height of the gravity meter, latitude and longitude of the GNSS, and antenna height of the GNSS receiver. This process is time-consuming and may require additional computing resources to convert relative gravity measurements into an absolute gravity value.

Advances in information and micro-electromechanical systems (MEMS) technologies can be used to solve the problems associated with conventional gravity measurements. In this study, we used a smartphone equipped with various sensors (e.g., complementary metal-oxide semiconductor (CMOS) camera, accelerometer, gyroscope) and various wireless communication devices (e.g., Wi-Fi, Bluetooth, third-generation (3G) or long-term evolution (LTE) devices) for network real-time kinematic (RTK) positioning and gravity surveying.

Despite their compact size, smartphones are equipped with processing and memory capabilities comparable to those of many personal computers (PCs) and mobile devices, such as personal digital assistants (PDAs), tablet personal computers (PCs), or other cellular phones. Additionally, based on 3G and LTE wireless networks, smartphones have the added benefit of being able to send and receive data and to connect to the Internet in real-time. As of November 2012, Google's Android operating system (OS) and Apple's iOS had ∼90% of the smartphone OS market share in the US (53.7% and 35.0%, respectively) [3]. Both of these companies provide a software development kit (SDK) and an integrated development environment (IDE) so that applications in their OS can be developed more easily.

Because smartphones are so easy to use, researchers can develop particular applications using the built-in sensors of the smartphone, even with little knowledge of hardware systems. As a result, smartphone use is on the rise in various fields including geology, medicine, education, and disaster preparedness and prevention. For example, reference [4] developed a seismometer app using the gyroscope sensor in a smartphone. Reference [5] evaluated the possibility of adopting a smartphone-based monitoring system for coastal monitoring through prevision evaluation using smartphone-based technology. In [6] an app making it possible to position network RTK using a smartphone was implemented. Educational content has been produced and distributed for plastic surgery trainees and surgeons with smartphones [7–9]. In [10] an Android phone application, GeoTools, to integrate essential tasks for geological field studies, including photography, videotaping, audio-recording, GNSS coordinate recording, measurements of strike and dip, note-taking, and data organization for the convenience of geologists was proposed. Realini, E. *et al.* [11] developed the Open Geospatial Consortium Web Processing Service (WPS) to process raw GPS observations and an RTK application to acquire 3-D positioning with sub-meter accuracy using a low-level GPS receiver in a lightweight device.

The purpose of this study was to develop a network RTK positioning and gravity-surveying app using a smartphone. The primary contributions of this study are summarized as follows:

1. Implementation of network-RTK-based GNSS positioning through a real-time connection between the GNSS receiver and a continuously-operating GNSS reference station (CORS) using wireless Internet.
2. Implementation of a function for gravity survey data input and management using gravimeter measurements.
3. Implementation of a module-enabling gravity correction (for tides, meter height, instrument drift corrections, and network adjustments) using gravity survey data that were collected along with the three-dimensional (3-D) position.
4. Implementation of functions to distribute results with gravity correction to other researchers via electronic mail (e-mail).

## 2. Experimental Section

### 2.1. Network RTK Based on a Mobile Device for 3-D Positioning

To determine the position of a mobile station, the RTK system sends and receives data between the CORS and the mobile station using a digital ultra-high frequency (UHF) communication link. The digital UHF model must be installed within its communication scope, so the RTK system has limited 3-D positioning capabilities.

Network RTK overcomes this weakness with centimeter-level accuracy, even at distances of tens of kilometers. The network RTK system, in which the reference station receiver is near a rover receiver, exhibits performance equivalent to that of a single-base RTK, to a maximum distance of 10 km [12]. Network RTK makes it possible to receive correction data created by the CORS, even from long distances, by adopting a code-division multiple-access/global system for mobile (CDMA/GSM) wireless communication networks instead of the existing digital UHF modem. Additionally, Network RTK sends data via networked transport of Radio Technical Commission for Maritime Services (RTCM) via Internet protocol (NTRIP).

NTRIP is an RTCM standard that is designed to distribute differential global positioning system (GPS) data-correction messages (e.g., in the RTCM-104 format) or other kinds of GNSS streaming data, based on Hypertext Transfer Protocol (HTTP). NTRIP consists of a source, server, caster and client. The NTRIP source normally indicates the GNSS receiver that provides observations or creates correction data. The NTRIP server forwards corrections from the reference station to the caster, and the caster (major broadcaster) forwards the major stream-splitting, broadcasting, and meta-data system components simultaneously to multiple users. The NTRIP client receives the desired source data, which are then applied to GNSS applications [13,14]. Commercially proven network RTK technologies include the Virtual Reference Station (VRS), area correction parameters (in German: Flächen-Korrektur-Parameter, FKP), the Master-Auxiliary Concept (MAC), and others [15].

In general, the network RTK system consists of a rover GNSS receiver, a wireless communication device for communication with the network RTK server, and the CORS. The operational mechanism is outlined as follows: (1) A rover GNSS receiver determines the user’ approximate position as GPS fixed data (GPGGA) (*i.e.*, GPGGA NMEA sentences with NMEA format) and then sends this to the CORS using a wireless communication device. (2) The virtual base station sends the corresponding RTK correction messages to the rover GNSS receiver in an RTCM format using the received position information. (3) The rover GNSS receiver then calculates the position using the received correction data. Feedback of the corrected value and its comparison allow a precise calculation of the position [16,17].

**Figure 1.**
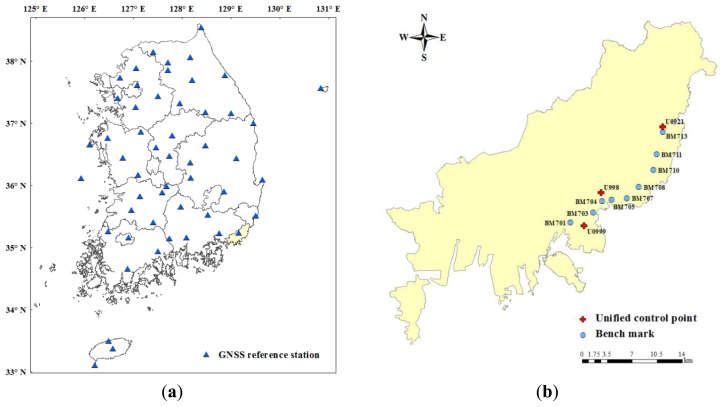
(**a**) Distribution of 52 GNSS reference stations in South Korea; (**b**) the study area (three unified control points (UCPs) and eight bench marks (BMs) used in the experiment).

Since April 2013, the National Geographic Information Institute (NGII) of South Korea has provided a GPS data-download service (http://gps.ngii.go.kr/) using information received by the 52 CORSs (Figure 1). This provides VRS and FKP data for use with network RTK technology.

### 2.2. Gravity Corrections

Gravity measurements were carried out using a relative gravimeter by calculating the absolute gravity based on the difference between each gravity station and a reference gravity station. The difference between each gravity station and the reference gravity station can be calculated following tidal corrections, height corrections, drift corrections, and network adjustments.

Tidal acceleration is caused by the superposition of lunisolar gravitation (and to a far lesser extent, planetary gravitation) and orbital accelerations due to the motion of the earth around the barycenter of each respective two-body system [2]. It can be calculated either from the ephemerides of celestial bodies or from a spherical harmonic expansion of the tidal potential. Reference [18] developed a computer program to compute tidal acceleration with a precision of 0.1 μGal, based on previous methods. All formulas in the program were expanded around the central epoch J2000.0, which retains its precision for the next 50 years. We used Tamura's Fortran program to compute the tidal acceleration.

**Figure 2.**
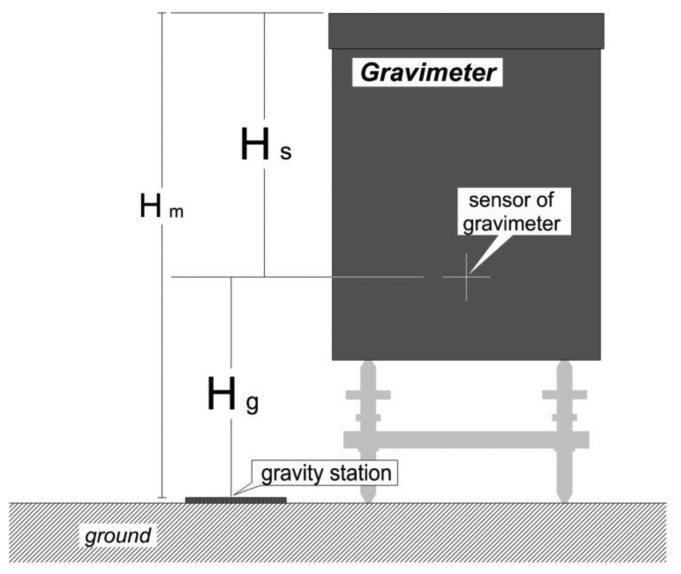
Graphical illustration of the meter height correction.

The location of a gravity anomaly is calculated as a coordinate, and the location of the gravity measurement is the gravimeter. The difference in height between two locations needs to be corrected for; this is termed the meter height correction. Figure 2 shows the height of gravimeter (*H_m_*), the difference in height between the upper part of gravimeter and the sensor (*H_s_*), and the difference in height between the gravimeter sensor and the gravity station (*H_g_*). *H_m_* is measured by an observer and *H_s_* can befoundwith reference to the manual. The height at which meter height correction is carried out is *H_g_*, which can be calculated using Equation (1). The meter height correction converts *H_g_* into a gravity change. In this case, a free-air gradient of −0.3086 mGal/m was applied:

(1) $$H_{g}=H_{m}-H_{s}$$

**Figure 3.**
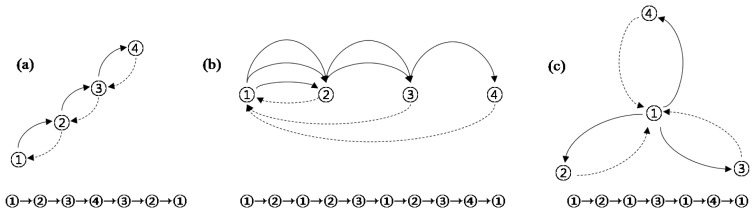
Drift determination methods: (**a**) profile method; (**b**) step method; and (**c**) star method (adapted from [2]).

The instrument-drift correction is used to correct for instrument drift that typically appears in an unstable gravity meter. This drift is caused by aging of the spring, reaction of the spring to vibration and shock during transport during a field survey, uncompensated temperature fluctuations, and elastic aftereffects following unclamping of the lever [2]. Drift correction is determined by examining the variation in gravity over time and taking repeated measurements for the same position. The profile method, star method, and step method can be used for this purpose (Figure 3); the particular method chosen depends on the characteristics of the gravity meter and the network [2].

The drift rate with respect to time is given as follows:

(2) $$\text{Drift Rate}=\frac{g_{2}-g_{1}}{t_{2}-t_{1}}$$

where *g*_1_ and *g*_2_ are the tide-corrected gravity values for the same stations at times *t*_1_ and *t*_2_, respectively. During the survey, the number of drift rates can be computed daily. These drift rates determine the daily drift rate (DDR) using least-squares fitting [18], the weighted mean, and the arithmetic mean. The drift-correction value computed at time *t* is expressed as:

(3) $$\text{Drift Correction Value}(t_{i})=\text{DDR}\times \frac{g_{t_{i}}-g_{t_{0}}}{t_{i}-t_{0}}$$

where *g_ti_* and *g_t_*_0_ are the gravity measurements at times *t_i_* and *t*_0_, respectively, where t_0_ corresponds to the first gravity measurement of the day.

The benefit of using the least-squares adjustment method is that statistical analysis can be carried out after adjustments are complete. The least-squares criterion is an imposed condition for obtaining a unique solution for an incompatible system of linear equations. Term adjustment, in a statistical sense, is a method of deriving estimates for random variables from their observed values. The application of the least-squares criterion in the adjustment problem is called the least-squares adjustment method [19]. We used the Cholesky decomposition method to compute absolute gravity using the algorithm in [20].

### 2.3. Smartphone-Based Application Design

**Figure 4.**
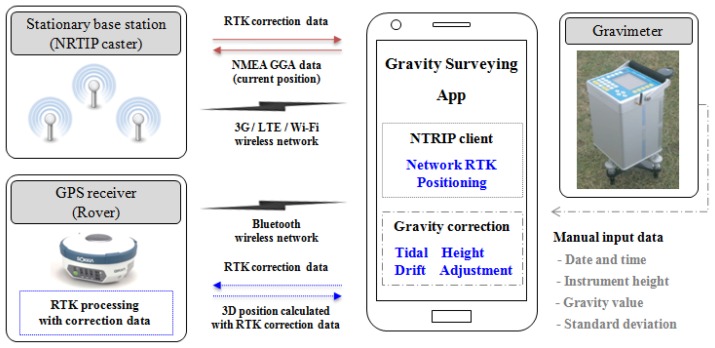
Application design overview consisting of NTRIP client and gravity correction.

The smartphone-based gravity-surveying app consists of a main module to manage and send the survey data, an NTRIP client module for network RTK-based positioning in the GNSS receiver, and a gravity-processing module for the tides, meter height, and instrument-drift corrections, as well as network adjustments (Figure 4). The proposed app downloads correction data by connecting to the CORS via the built-in wireless communication devices (Bluetooth and 3G or LTE) in the smartphone, and sends it to the GNSS receiver. Thus, the app provides precise, real-time positioning information using network RTK technology.

The app stores the measured gravity from the gravimeter. The user-friendly environment provided by the app allows for corrections to the measured gravity values using geoid models (e.g., the EGM2008 model (EGM2008*_NGA_*) proposed by the National Geospatial Intelligence Agency (NGA) Earth Gravitational Model (EGM) development team, and the fitted EGM2008 model (EGM2008*_fitted_* proposed by [21]) for computing orthometric height (hereafter ‘height’), drift-correction models (e.g., arithmetic mean, weighted mean, and least-squares fitting), user-defined NTRIP caster servers, and projections.

The main module stores 3-D position data (latitude, longitude, and ellipsoidal height) and gravity obtained from the GNSS receiver and the relative gravimeter in an SQLite database and sends the data processed by the gravity-processing module via email. The network RTK-based positioning module connects to the GNSS receiver and the NTRIP caster of the CORS via 3G or LTE network. The user's approximate position data and RTK correction messages are sent from the GNSS receiver to the CORS. The 3-D position, calculated from the data received, is then used to determine the height from the built-in geoid height data in the smartphone. The gravity-processing module also provides gravity-correction functions for tide data, meter height, instrument-drift corrections, and network adjustments. The corrected results are then stored in the SQLite database.

The NTRIP client uses a GNSS receiver interface language (GRIL) to control the GNSS receiver. After a smartphone is connected to the GNSS receiver and an NTRIP caster, the procedure for 3-D positioning using network RTK technology from GNSS receiver is as follows. First, the NTRIP client sets the network RTK mode at the GNSS receiver and then sends a command to acquire the position data and NMEA GGA data. Next, the GNSS receiver sends the position messages calculated without the RTK correction data and the NMEA GGA data to the NTRIP client. Next, the NTRIP client transfers the data from the GNSS receiver to the message parser. From these data, the message parser delivers the GGA data to the NTRIP client, while it delivers position data to users. Next, the NTRIP client that received the GGA data sends a message to the NTRIP caster and requests the RTK correction data. Next, the NTRIP caster streams the RTK correction data to the NTRIP client, and the NTRIP client streams the received RTK correction data to the GNSS receiver. The GNSS receiver streams precise position data calculated using network RTK technology to the NTRIP client.

### 2.4. Experimental Setup

This study conducted network RTK positioning and gravity surveys at eight bench mark (BM) points and three unified control points (UCP) in Busan, South Korea to evaluate the performance of the developed app. The equipment used in the experiment included the GRX1 GNSS receiver (Sokkia, Tokyo, Japan) and a CG-5 gravimeter (Scintrex, ON, Canada) (Table 1).The network RTK positioning used correction data with a duration of 1 second, which was provided by NGII. Gravity was measured using the profile method, considering the spatial layout of the points in research areas, as shown in Figure 1b.

## 3. Results and Discussion

### 3.1. Smartphone-Based Application Development

In this study, a smartphone-based gravity survey app was developed using an Android Software Developer's Kit (Android SDK) so that it could run on an Android OS-based smartphone. Specifically, the app used an Android OS 2.2 configuration, with Android API Level 15 SDK targeted for Android OS 4.03 (Table 2).

The app was also implemented using Java DK 1.6 and Eclipse IDE for JAVA developers. GIRL was used to control the GNSS receiver. The height value specified by network RTK positioning was an ellipsoidal height. A geoid height value was required to determine the orthometric height; in this study, orthometric height was calculated using EGM2008*_fitted_*, which provides a more accurate geoid height of the Korean Peninsula using GPS leveling data, and is based on the EGM2008*_NGA_*.

**Figure 5.**
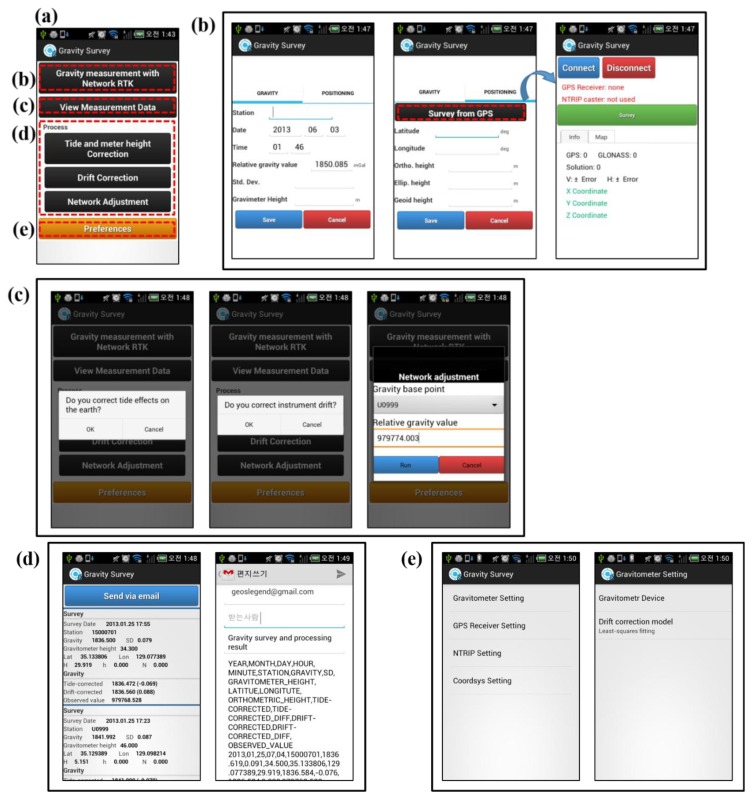
Screen shots of the gravity survey app: (**a**) main screen; (**b**) gravity and 3-D position measurement with the network RTK technology screen; (**c**) tide, meter height, and drift corrections, as well as network adjustment; (**d**) viewing measured data and results (with 3-D position and survey values) and sending them via email; and (**e**) environmental settings.

The app consists of gravity measurement using network RTK, a viewing facility for the measured data and results, and process and preferences screens, as shown in Figure 5a. The ‘gravity measurement with network RTK’ screen allows collection of gravimetric data, measured by the gravimeter, and 3-D positioning is enabled via RTK technology in the receiver's network (see Figure 5b). The process group consists of tide, meter height, and drift corrections, as well as network adjustment, making it possible to carry out gravity correction (see Figure 5c). Here, the reference gravity station and relative gravimetric data on the station are required to carry out network adjustment. The ‘view measured data and results screen’ shows the gravimetric data and the 3-D position measured from the ‘gravity measurement with network RTK’ screen, as well as the gravity correction results. This screen also provides a function to send data via e-mail to researchers (see Figure 5d). The ‘preferences’ screen enables the user to set environmental variables for the gravity survey app, including the gravimeter, GPS receiver, NTRIP server, and coordinate system settings. Arithmetic mean, weighted mean, and least-squares fitting in the drift correction model can be selected.

Gravity and 3-D position data can be observed using the app as follows: select ‘preferences’ before observation, then select ‘gravity measurement with network RTK’ in the main screen of the app. In the gravity tab, input the station name, gravimeter height and date, time, relative gravimetric value, and standard deviation observed by gravimeter. In the positioning tab, directly input the latitude, longitude, orthometric height, ellipsoidal height, and geoid height or observe these using ‘survey from GNSS receiver,’ then enter these data automatically.

The observed data can be corrected using the tide and meter height correction, drift correction, and network adjustment in the process group. The observed data and correction results can be viewed by selecting ‘view measured data and results’ in the main screen. The data can be added as an attachment to an e-mail in a comma-separated value (CSV) file format.

### 3.2. Experiment Results

Network RTK positioning and gravity surveying experiments were conducted with the proposed app to evaluate its performance. We encountered some difficulty in obtaining 3-D positioning data for the three UCPs and eight BMs due to interference from trees. Therefore, the experiment instead used the coordinates of the control-point data provided by NGII, specifically three BMs: BM701, BM705, and BM707 (the symbol ‘-’ is omitted). Network RTK positioning was performed for UCP U0999 for ∼1 h using the proposed app with EGM2008*_fitted_*. The measured data were then compared with the control-point data provided by NGII.

**Figure 6.**
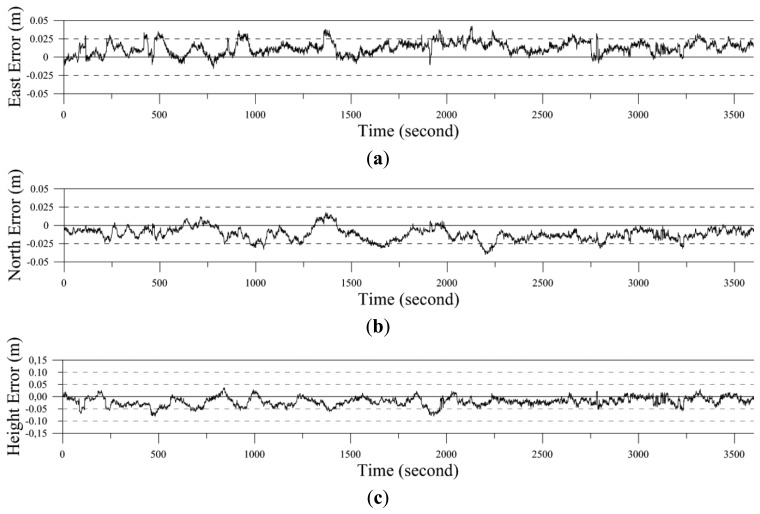
Accuracy test results at UCP U0999 in network RTK positioning: (**a**) position error for east coordinates; (**b**) position error for north coordinates; and (**c**) position error for height coordinates.

Table 3 and Figure 6 show the results of positioning errors for the north and east directions and for height. The differences in the measurements ranged from −0.040 to 0.018 m in latitude, −0.016 to 0.042 m in longitude, and −0.081 to 0.037 m in height. Results of network RTK positioning from other research [23] has indicated a horizontal positioning accuracy of ±5 cm in Southern Germany. [6,12,24] demonstrated positioning errors of ±2.0 cm and ±5.0 cm for the horizontal and height coordinates, respectively, using network RTK. It follows that the NTRIP client in the app can carry out 3-D positioning using network RTK technology easily and in real-time using the GNSS receiver.

The role of the NTRIP client is to carry out network RTK-based 3-D positioning in real-time at the GNSS receiver. The NTRIP client receives correction data from the CORS and sends it to the GNSS receiver. Unless the correction data is sent in real-time, the accuracy of the 3-D positioning at the GNSS receiver rover is degraded. Additionally, the positioning accuracy in the Network RTK can vary depending on the observation area, GNSS status of the CORS, geological layout of the reference station, and the length of the baseline.

**Figure 7.**
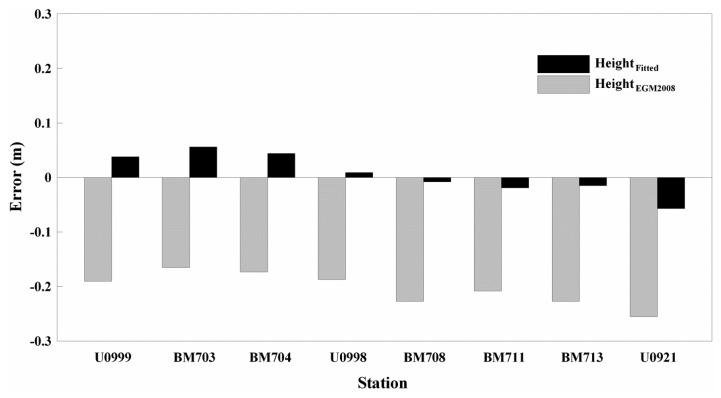
Height accuracy evaluation for Network RTK positioning using EGM2008*_NGA_* and EGM2008*_Fitted_* applied to the smartphone.

To evaluate the accuracy of the Height*_Fitted_*, calculated by the geoid height of EGM2008*_Fitted_* and applied to the network RTK positioning of the proposed app, this study compared Height*_EGM2008_* calculated from EGM2008*_NGA_* with the Height*_ControlPoint_* of the control point data (Figure 7).

The difference results for Δ(Height*_ControlPoint_* − Height*_EGM2008_*) ranged from −0.255 to −0.165 m, with an average difference of −0.204 and a standard deviation of 0.031. Δ(Height*_ControlPoint_* − Height*_Fitted_*) ranged from −0.057 to 0.056 m, with an average difference of 0.006 m and a standard deviation of 0.038 m. However, the vertical accuracy was lower for Δ(Height*_ControlPoint_* − Height*_EGM2008_*) than for Δ(Height*_ControlPoint_* − Height*_Fitted_*). This finding suggests that the coordinates of the gravity stations using the height calculated from EGM2008*_Fitted_* may provide better accuracy.

The measured gravity values were corrected for tides, meter height, and instrument drift, as well as network adjustments using the built-in gravity-processing module in the proposed app (Table 4, Appendix A). The CG-5 relative gravimeter used in this study required a reference gravity station to calculate an absolute gravity value. This allowed the measured gravity data to be converted to a value for absolute gravity. UCP U0999 was used for the reference gravity station.

Our experimental results indicated a terrestrial tide correction ranging from −0.083 to −0.002 mGal and a meter height correction of 0.039 to 0.077 mGal. The standard deviation for drift correction was 0.040 mGal·h^−1^ for the arithmetic mean, 0.039 mGal·h^−1^ for the weighted mean, and 0.037 mGal·h^−1^ for least-squares fitting. Among the correction methods, least-squares fitting yielded the best results and was used for network adjustments. The correction for drift was 0.004 to 0.088 mGal·h^−1^. Gravity values after tide, meter height, and drift correction indicated −0.082 to 0.035 mGal difference compared with the initial observation value. Table 4 shows the absolute gravity values calculated from the corrected gravity values. The reference standard deviation was 0.038 mGal after network adjustment, which was lower than the 0.065–0.125 mGal standard deviation obtained from the relative gravimeter (Appendix A).

## 4. Conclusions

We have described the development of a network RTK positioning and gravity-surveying app using a smartphone equipped with built-in Bluetooth and a 3G (or LTE) chip. The proposed app provided an operational environment for network RTK positioning and gravity surveying. The gravity-processing module of the app converted relative gravity values into absolute gravity. Additionally, corrections to the gravity measurements due to errors associated with tides, meter height, instrument drift, and network adjustments could be made in semi-real-time. The instrument-drift correction module optimized the accuracy of the difference measurements using least-squares fitting, the weighted mean, and the arithmetic mean.

To evaluate the performance of the proposed app, network RTK positioning and a relative gravity survey were carried out using eight points (five BMs and three UCPs) and eleven points (eight BMs and three UCPs).The results of network RTK positioning at UCP U0999 revealed difference ranges of −0.016 to 0.042 m for the north direction and −0.016 to 0.042 m for the east, with a height variation of −0.081 to 0.037 m. The observed relative gravity was calculated as an absolute value with tide, meter height, and drift corrections, and network adjustment procedure. The reference standard deviation after network adjustment was 0.038 mGal. Together, our results suggest that gravity-survey data can be processed in semi-real-time in the field using the proposed app. This will allow researchers to share and compare results more efficiently. Additionally, the proposed system for the app is compact and easily installed for 3-D positioning and gravity measurements.

## Figures and Tables

**Table 1.** Equipment specifications [1,22].

	**Sokkia GRX1 GNSS Receiver**		**Scintrex CG-5**
Positioning accuracy			
Static L1 + L2	H: 3 mm + 0.5 ppm	Sensor type	Fused Quartz using electrostatic nulling
	V: 5 mm + 0.5 ppm		
L1 only	H: 3 mm + 0.8 ppm	Reading resolution	1 microGal
	V: 4 mm + 1 ppm		
Fast static L1 + L2	H: 3 mm + 0.5 ppm	Standard field repeatability	<5 microGal
	V: 5 mm + 0.5 ppm		
Kinematic L1 + L2	H:10 mm + 1 ppm	Operating range	8,000 mGal without resetting
	V:15 mm + 1 ppm		
RTK L1 + L2	H:10 mm + 1 ppm	Residual long-term drift	Less than 0.02 mGal/day(static)
	V: 15 mm + 1 ppm		
DGPS	<0.5 m		

**Table 2.** Android OS-based app development environment.

**Item**	**Detail**
Operation System	Android OS 4.03
SDK	Android API Level 15 with Google APIs & JDK 1.6
IDE	Eclipse IDE for Java Developers Indigo
Programming Language	Java
Command interpreter language of GNSS Receiver	GNSS receiver interface language

**Table 3.** Statistical table for positioning errors at U0999 in network RTK positioning (unit: meter).

	**North**	**East**	**Height***_Fitted_*
Minimum	−0.016	−0.040	−0.081
Maximum	0.042	0.018	0.037
Average	0.013	−0.012	−0.020
Standard deviation	0.009	0.009	0.019

**Table 4.** Absolute gravity values after network adjustment correction.

**Station**	**Latitude (deg)**	**Longitude (deg)**	**Height (m)**	**Absolute Gravity value (mGal)**
U0999 a	35.129389	129.098214	5.151	979,774.003
BM701	35.133806	129.077389	29.919	979,768.528
BM703	35.145540	129.112424	18.701	979,771.663
BM704	35.160045	129.126843	18.956	979,770.424
BM705	35.161306	129.141083	3.934	979,775.572
U0998	35.170715	129.125532	5.474	979,773.292
BM707	35.163056	129.164194	5.391	979,773.306
BM708	35.176490	129.183252	49.806	979,764.768
BM710	35.197722	129.205833	5.520	979,777.747
BM711	35.217256	129.211315	56.904	979,768.769
BM713	35.245010	129.221552	17.816	979,778.458
U0921	35.251694	129.222266	23.664	979,776.965

a Reference gravity station.

## Appendix A

**Table:** Gravity survey data and the results after tide and drift corrections.

**Survey Date**	**Station**	**Relative Gravity Value (mGal)**	**SD (mGal)**	**Meter Height (m)**	**Latitude (deg)**	**Longitude (deg)**	**Height (m)**	**Tide-Corrected (mGal)**	**Diff (mGal)**	**Meter-Corrected (mGal)**	**Diff (mGal)**	**Drift-Corrected (mGal·h^−1^)**	**Diff (mGal·h^−1^)**
2013-01-25 07:04	BM701	1,836.619	0.091	34.5	129.077389	35.133806	29.919	1,836.543	−0.076	1,836.584	0.041	1,836.584	0.000
2013-01-25 07:30	U0999	1,842.012	0.071	45.5	129.098213	35.129390	5.113	1,841.942	−0.070	1,842.017	0.075	1,842.021	0.004
2013-01-25 07:53	BM703	1,839.698	0.065	35.0	129.112424	35.145540	18.701	1,839.635	−0.063	1,839.678	0.043	1,839.684	0.007
2013-01-25 08:16	BM704	1,838.446	0.089	35.0	129.126843	35.160045	18.956	1,838.391	−0.055	1,838.434	0.043	1,838.443	0.010
2013-01-25 08:39	BM705	1,843.592	0.084	37.0	129.141083	35.161306	3.934	1,843.545	−0.047	1,843.594	0.049	1,843.607	0.013
2013-01-25 09:06	U0998	1,841.291	0.084	45.5	129.125532	35.170715	5.474	1,841.255	−0.036	1,841.330	0.075	1,841.347	0.017
2013-01-25 09:36	BM707	1,841.284	0.084	35.3	129.164194	35.163056	5.391	1,841.259	−0.025	1,841.303	0.044	1,841.324	0.021
2013-01-25 10:13	BM708	1,832.722	0.088	34.4	129.183252	35.176490	49.806	1,832.709	−0.013	1,832.750	0.041	1,832.776	0.026
2013-01-25 10:45	BM710	1,845.709	0.100	34.6	129.205833	35.197722	5.520	1,845.703	−0.006	1,845.745	0.042	1,845.775	0.030
2013-01-25 11:16	BM711	1,836.707	0.122	35.4	129.211315	35.217256	56.904	1,836.705	−0.002	1,836.749	0.044	1,836.783	0.034
2013-01-25 11:50	BM713	1,846.405	0.12	34.0	129.221552	35.245010	17.816	1,846.402	−0.003	1,846.442	0.040	1,846.481	0.039
2013-01-25 12:12	U0921	1,844.950	0.101	35.9	129.222266	35.251694	23.664	1,844.945	−0.005	1,844.991	0.046	1,845.032	0.042
2013-01-25 13:13	U0921	1,844.912	0.111	35.9	129.222266	35.251694	23.664	1,844.891	−0.021	1,844.937	0.046	1,844.987	0.050
2013-01-25 13:40	BM713	1,846.461	0.099	33.6	129.221552	35.245010	17.816	1,846.431	−0.030	1,846.469	0.039	1,846.523	0.054
2013-01-25 14:02	BM711	1,836.783	0.125	35.2	129.211315	35.217256	56.904	1,836.744	−0.039	1,836.788	0.044	1,836.844	0.057
2013-01-25 14:19	BM710	1,845.754	0.093	34.5	129.205833	35.197722	5.520	1,845.708	−0.046	1,845.749	0.041	1,845.808	0.059
2013-01-25 14:36	BM708	1,832.800	0.107	34.4	129.183252	35.176490	49.806	1,832.747	−0.053	1,832.788	0.041	1,832.850	0.061
2013-01-25 15:05	BM707	1,841.332	0.081	35.3	129.164194	35.163056	5.391	1,841.268	−0.064	1,841.312	0.044	1,841.377	0.065
2013-01-25 15:41	U0998	1,841.283	0.088	36.4	129.125532	35.170715	5.474	1,841.208	−0.075	1,841.255	0.047	1,841.325	0.070
2013-01-25 16:06	BM705	1,843.583	0.089	37.1	129.141083	35.161306	3.934	1,843.503	−0.080	1,843.552	0.049	1,843.625	0.073
2013-01-25 16:27	BM704	1,838.456	0.080	35.1	129.126843	35.160045	18.956	1,838.373	−0.083	1,838.416	0.043	1,838.492	0.076
2013-01-25 16:57	BM703	1,839.690	0.079	35.0	129.112424	35.145540	18.701	1,839.607	−0.083	1,839.650	0.043	1,839.730	0.080
2013-01-25 17:23	U0999	1,841.992	0.087	46.0	129.098213	35.129390	5.113	1,841.913	−0.079	1,841.990	0.077	1,842.074	0.084
2013-01-25 17:55	BM701	1,836.500	0.079	34.3	129.077389	35.133806	29.919	1,836.431	−0.069	1,836.472	0.041	1,836.560	0.088
